# The protein elicitor Hrip1 enhances resistance to insects and early bolting and flowering in *Arabidopsis thaliana*

**DOI:** 10.1371/journal.pone.0216082

**Published:** 2019-04-25

**Authors:** Xin-yue Miao, Hong-pan Qu, Ya-lei Han, Cong-fen He, De-wen Qiu, Zhi-wei Cheng

**Affiliations:** 1 Beijing Key Laboratory of Plants Resource Research and Development, School of Sciences, Beijing Technology and Business University, Beijing, China; 2 Aerospace Center Hospital, Cardiovascular Department, Beijing, China; 3 State Key Laboratory for Biology of Plant Diseases and Insect Pests, Institute of Plant Protection, Chinese Academy of Agricultural Sciences, Beijing, China; National Taiwan University, TAIWAN

## Abstract

The elicitor Hrip1 isolated from necrotrophic fungus *Alternaria tenuissima*, could induce systemic acquired resistance in tobacco to enhance resistance to tobacco mosaic virus. In the present study, we found that the transgenic lines of *Hrip1*-overexpression in wild type (WT) *Arabidopsis thaliana* were more resistant to *Spodoptera exigua* and were early bolting and flowering than the WT. A profiling of transcription assay using digital gene expression profiling was used for transgenic and WT *Arabidopsis thaliana*. Differentially expressed genes including 40 upregulated and three downregulated genes were identified. In transgenic lines of *Hrip1-*overexpression, three genes related to jasmonate (JA) biosynthesis were significantly upregulated, and the JA level was found to be higher than WT. Two GDSL family members (*GLIP1* and *GLIP4*) and pathogen-related gene, which participated in pathogen defense action, were upregulated in the transgenic line of *Hrip1*-overexpression. Thus, Hrip1 is involved in affecting the flower bolting time and regulating endogenous JA biosynthesis and regulatory network to enhance resistance to insect.

## Introduction

Plants are constantly adapting to environmental changes of abiotic and biotic stresses, because of their evolved ability to appropriately respond to changes in stressful conditions [1–4]. The innate immune systems of plants detect the invasion signals’ response to biotic stress and initiate the regulation of plant growth and defense [3]. Some effectors including plant phytohormones, pathogenesis-related protein (PR), and GDSL-type esterases/lipases play critical roles to mediate these regulatory signaling networks.

Phytohormones are produced in the plants and they control the behavior of growth and defense in plants. Jasmonates (JAs), a set of fatty acid-derived signaling molecules, are involved in many developmental processes, such as root growth, tuberization, tendril coiling, pollen development, and seed germination [5]. They are also involved in plant response to environmental stresses, such as water deficit, ozone exposure, pathogen infection, wounding, and pest attack [6–16]. The biosynthesis of JA in plants starts from *α*-linolenic acid (18:3) in chloroplast membranes [15, 17]. Subsequently, *α*-linolenic acid is catalyzed by a sequence of biosynthesis enzymes, including 13-lipoxygenase (LOX), allene oxide synthase (AOS), and allene oxide cyclase (AOC), to produce OPDA in plastid [15, 17–20]. OPDA was imported into peroxisome by PXA1, an ATP-binding cassette transporter, and catalyzed by a series of enzymes that included OPR3 (OPDA reductase), OPCL1 (OPC-8:0 CoA ligase), five acyl-CoA oxidases (ACX1–ACX5), and MFP (multifunctional protein) to yield (+)-7-iso-JA [15, 21, 22]. In cytosol, (+)-7-iso-JA is conjugated with the amino acid isoleucine (Ile) to synthesize JA-Ile, which is known as a bioactive form of JAs [23]. The JA receptor coronatine insensitive 1 (COI1) [24–26] recruits JA-ZIM-domain proteins (JAZs) [27, 28] for ubiquitination and degradation by 26S proteasome, when JA-Ile is recognized by COI1 in plants [27–29]. The transcription factors (such as MYC2) which are repressed by JAZs, are released to launch the expression of JA-response gene and initiate JA-regulated functions including JA-inhibitory root growth [30], plant fertility [30, 31], and resistance against pathogens and insects [32].

The plants have two modes of immunity: pathogen-associated molecular pattern-triggered immunity (PTI) and effector-triggered immunity (ETI). The pathogen-associated molecular patterns derived from the components of the structures microorganisms or pathogens, which are recognized by pattern recognition receptors of plants, induce the further activation of PTI [33]. The secret effector protein of microbial pathogens, which are distinguished by resistance (R) protein, initiates the activation of the ETI [34]. To date, many PR proteins that directly interact with identified pathogen effector proteins have been widely recognized [35]. The PR proteins are absent or present at low concentrations in healthy plants, but they are induced and accumulated at protein level during pathological infection (such as fungi, bacteria, viruses, insects, and herbivores) and related situations including the application of phytohormones and wounding [36–39]. Furthermore, PR proteins are low-molecular-weight proteins (5–75 kDa), which are thermostable, resistant to proteases, and able to dissolve at low pH (<3) [36, 39]. Presently, PR proteins are divided into 17 families that are based on protein sequence features, enzymatic activities, and other biological functions [36].

Some PR proteins (PR-6, PR-12, PR-13, and PR-14) with a molecular size below 10 kDa are defined as PR peptides or antimicrobial peptides, which are cysteine-rich molecules and play an important role in host resistance against microbial pathogens and pests in plants [36, 39, 40]. PR-6 peptides belong to a subclass of serine proteinase inhibitors (PIs), which are similar to “tomato/potato inhibitor I” [36, 41]. PIs are usually able to bind proteinases and control proteinase activity; they play a range of roles in defense, including weakening the ability of an attacker to (i) obtain lytic enzymes to resist fungi [42], (ii) disturb viral replication cycles [43], and(iii) act against the digestive protease used by nematodes and insects [44–46]. Numerous articles have reported that transgenic plants with heterologous PI gene overexpression enhance the resistance of plants to insect attack [47]. *Arabidopsis* has six *PI* genes encoding the PR-6-type protein, and the calculated isoelectric point (pI) of the predictive protein ranges from 4.6 to 11.3 [36]. A PR-6 protein (At2g38870) is overexpressed in *Arabidopsis*, and transgenic plants have the ability to enhanced resistance to *Botrytis cinerea* [48].

GDSL-type esterases/lipases, a type of lipid hydrolysis enzyme, are common in bacteria and plants including rice, maize, and *Arabidopsis*; they have multifunctional properties [49]. In *Arabidopsis*, 105 GDSL-type esterase/lipase (AtGELP) genes have been identified; these genes were divided into four clades depending on their functions related to morphological development, abiotic stress response, secondary metabolism, and pathogen defense [49]. In clade IIIa of AtGELP, several proteins exhibit functions related to biotic responses, such as AtGELP97 (GLIP1), ATGELP20 (GLIP2), AtGELP63 (ESM), and *BrSil* [49]. GLIP1 [50] regulates plant immunity via the ethylene signaling pathway, which upregulates the expression of *ETHYLENE RESPONSE FACTOR*1 (*ERF1*) and represses the expression of *ETHYLENE INSENTSTIVE 3* (*EIN3*) [51, 52]. Furthermore, the overexpression of *GLIP1* in *Arabidopsis* increases the expression of *SALICYLIC ACID INDUCTION-DEFICIENT2* (*SID2*), a salicylic acid biosynthesis gene [51]. The recombinant GLIP2 protein exhibits lipase and antimicrobial activities, resulting in resistance to *Erwinia carotovora* (*Pectobacterium carotovora*). The *Arabidopsis* T-DNA insertion mutant of *glip2* is more susceptible to *E*.*carotovora* and manifests enhanced auxin response, which indicates that GLIP2 negatively regulates auxin signals to participate in pathogen defense in plants [53].

Protein elicitors, such as Harpin protein, flagellin, elicitin, activator, and glycoprotein [54, 55], are involved in both biotic and abiotic stress responses and trigger systemic acquired resistance (SAR) in plants infected by pathogens [56–58]. The Harpin protein has been isolated from *Erwinia amylovora*, and it is reported to trigger pathogen resistance in plants [59]. Transgenic tobacco plants with Harpin overexpressing exhibited phenotype resistance to pathogen infection [60, 61], and the expression of some genes is induced via the defense-related signaling pathway (generating nitric oxide [NO] and JA signaling pathway)[62]. MoHrip1 was purified from the extraction of *Magnaporthe oryzae*, which triggers the tobacco defense response, induces the expression of PR genes, and enhances systemic resistance to *M*.*oryzae* in rice seedlings [63]. The protein elicitor PevD1 isolated from V*erticillium dahliae* can enhance resistance to pathogen infection in plants, metabolite deposition, and cell wall modification [64, 65]. Transgenic lines with PevD1 overexpressing are highly resistant to *B*.*cinerea* and *Pseudomonas syringae pv*. *Tomato* DC3000[66].

Hrip1 is a novel elicitor that was purified from the necrotrophic fungus *Alternaria tenuissima* [67]. The protein Hrip1 comprises 163 amino acid residues, which are encoded by a 495 bp open reading frame (GenBank accession number HQ713431). In our previous work, the results indicated that Hrip1 triggers the hypersensitive response, generates necrotic lesions, and induces SAR in tobacco leaves that were inoculated with mosaic virus [67]. Furthermore, the transgenic lines of Hrip1 in *Arabidopsis* were more resistant to stresses and exerted a significant effect on plant height, silique length, and plant dry weight under the conditions of salt and drought compared with the WT [68]. In this study, *Hrip1* was transferred into the *Arabidopsis* genome by *Agrobacterium tumefaciens*. We investigated the bolting time and pathogen response phenotypes in transgenic plants distinguished from the WT. We also used high-throughput RNA-seq digital gene expression profiling (DGE) to explore the significant differential expression of genes in transgenic lines compared with WT. Our results furnished reliable information to facilitate our understanding of functions and mechanisms, including how Hrip1 regulated pathogen resistance and development in plants.

## Materials and methods

### Plant growth environment

Seeds including WT *Arabidopsis thaliana* (*Col-0*) and transgenic plants (35:*Hrip1*) were surface-sterilized with 10% bleach plus 0.1% tween-20 for 15 min, washed using sterile water for more than five times, and placed on growth medium (Murashige and Skoog, MS). The plates were transferred in a growth chamber (Percival AR800, USA) for 7 days until the seeds were germinated contained two euphylla. All plantlets grown in nutrient soil were grown in the growth chamber with 16 hrs light/8 hrs dark cycle at 22°C and 60% relative humidity.

### Vector construction and plant transformation

A truncated *Hrip1* gene without a signal peptide was amplified with its special primer and cloned into the *Nco* I and *BstE* II sites of the *pCAMBIA*1301 vector. This recombinant vector was transformed into *A*.*tumefaciens* strain *LBA4404*. The floral dip method was employed to complete the *Agrobacterium*-mediated transformation of *Col-*0 [69]. The T1 seeds were collected from transformed *Col-0* (T0 generation) and then generated in screening medium supplemented with 25 μg/mL hygromycin. Hygromycin-resistance T1 seedlings were transplanted into soil to harvest the T2 seeds. The T2 seeds were screened using hygromycin and selected in accordance with Mendel’s law. Six independent T3 transgenic lines were homozygous and used in our experiments.

### Real-time PCR

Total RNA was isolated using TRIzol reagent (Invitrogen, Carlsbad, California, CA, USA) and reverse-transcribed into cDNA using the kit (TransScript All-in-One First-Strand cDNA Synthesis SuperMix, TransGen Biotech) according to the manufacturer’s protocol. Quantitative RT-PCR (qRT-PCR) was performed using Power SYBR Green Master Mix (Invitrogen) and an iQ5 real-time PCR instrument (Bio-Rad Laboratories). Reaction system and amplification protocol referred to the manufacturer’s introductions. *ACTIN2* and *ACTIN7* were used as normalizing controls. All primers were designed by online-tools Universal ProbeLibrary Assay Design Center (Roche, https://lifescience.roche.com/en_cn/brands/universal-probe-library.html#assay-design-center) for qRT-PCR. The result of each qRT-PCR reaction was repeated at least three times.

### Western blot analysis

The leaves were collected and ground into powder in liquid nitrogen after the plants were grown in a growth chamber for 10 days. The powder was mixed well with extraction buffer (20 mM Tris-HCl pH 8.0, 20 mM NaCl, 1 mM EDTA, 1 mM phenylmethylsulfonyl fluoride [PMSF], and 1×ProteinSafe Protease Inhibitor Cocktail (100×) [TransGen Biotech]) and incubated for 30 min under ice block. The mixer was centrifuged at 20,000 g for 30 min at 4°C, and the supernatant was retained and used as crude protein. The boiled protein samples were electrophoresed using 15% SDS-PAGE and transferred onto PVDF membranes by the wetting transfer method. Immunoblotting was conducted using Anti-Hrip1 rabbit polyclonal antibodies (prepared by our lab) [67] or Anti-Actin Mouse Monoclonal Antibodies (TransGen Biotech, CAT: HC201) and ProteinFind Goat Anti-Rabbit IgG (H+L) with HRP conjugate (TransGen Biotech, CAT: HS101) or ProteinFind Goat Anti-Mouse IgG (H+L) with HRP conjugate (TransGen Biotech, CAT: HS201). The specific protein signals after immunoblotting were detected using photographic film under routine operation. These results were repeated more than three times.

### Detection of H_2_O_2_ assay

Detection of H_2_O_2_ assay was performed as previously described [70]. Seeds including WT *Arabidopsis thaliana* (*Col-0*) and transgenic plants (35:*Hrip1*) were surface-sterilized, and placed on MS medium to germination and growth. The leaves were detached from *Arabidopsis* which were grown MS medium for 7 days to 14 days (the days started from seed germination), was infiltrated with a solution of 1 mg/L diaminobenzidine dissolved in water. Leaves were placed on 3MM filter paper, then fixed with a solution of ethanol:lactic acid:glycerol (3:1:1, v/v/v), washed with 75, 50, and 25% ethanol, equilibrated with water, and photographed[70].

### Insect defense assay

Insect defense assay was performed as previously described [71]. The second-instar larvae of *S*.*exigua* were purchased from Henan Jiyuan Baiyun Industry Co., Ltd.. The larvae were transferred in plastic Petri dishes (150 mm) containing 1.5% phytagel and fed with 30 rosette leaves (these leaves were replaced with fresh leaves every 2 days from 2-week-old plants growing on nutrient soil) from WT or *Hrip1* transgenic plants. The larvae were fed for 6 days in the plastic Petri dish [71]. The larvae were weighed at 6 days after feeding using ten independent replicates. Student’s *t*-test was employed to determine statistically significant differences compared with the WT (*p < 0.05; **p < 0.01). These results were repeated at least three times.

### Bolting assay of transgenic lines

The seedlings of each genotype were germinated on MS medium for 1 week and then transferred into nutrient soil. After WT and transgenic plants were grown for 10 days under a growth chamber, the bolting phenotype was observed and recorded daily. Flowering time was measured by scoring the number of rosette leaves at the time of bolting. The data measured from 30 independent lines were analyzed with Student’s *t*-test, and asterisks indicate statistically significant differences compared with WT (*p < 0.05; **p < 0.01). All the plants were grown in the same growth chamber under the same conditions (16 hrs light/8 hrs dark cycle at 22°C with 60% relative humidity). These results were repeated more than three times.

### RNA-sequence with DGE

The *Arabidopsis* leaves (before bolting of plants grown for two-week-old on nutrient soil) were ground into a powder under liquid nitrogen, and total RNA was isolated from powders of plants using TRIzol reagent (Invitrogen, Carlsbad, CA, USA) following the protocol provided by the manufacturer. The quality of total RNA was monitored on 1% agarose gels and checked by NanoPhotometer Spectrophotometer (IMPLEN, CA, USA). The quantity of total RNA of each samples was measured using Qubit RNA Assay Kit in a Qubit 2.0 Fluorometer (Life Technologies, CA, USA). RNA integrity was evaluated by RNA Nano 6000 Assay Kit in a Bioanalyzer 2100 system (Agilent Technologies, CA, USA).

Approximately 3 μg of RNA from each sample was used to construct DGE libraries, which were made by NEBNext Ultra RNA Library Prep kit for Illumina (NEB) following the manufacturer’s recommendations. The preferential cDNA fragment (library fragments) whose length was 150–200 bp was collected using an AMPure XP system (Beckman Coulter, Beverly, CA, USA). The library fragments were amplified by PCR with Phusion High-Fidelity DNA polymerase, universal PCR primers, and an index (X) primer. The PCR products were purified (AMPure XP system), and library quality was appraised using an Agilent Bioanalyzer 2100 system. The index-coded samples were clustered on a cBot Cluster Generation System using a TruSeq PE Cluster Kit v3-cBot-HS (Illumina) in accordance with the manufacturer’s instructions. The sequence of each library fragments was read by an Illumina HiSeq 2000 platform.

### Analysis of DGE sequence results

Raw reads processed by removing reads containing adapters, reads containing poly-N, low quality reads, and cleaned reads were prepared for further analysis. Index of the *Arabidopsis* genome established by Bowtie v2.0.6 and single-end reads were matched to the reference genome using TopHat v2.0.9. The Reads Per Kilobase of exon model per million (RPKM) of each gene was calculated by HTSeq v0.6.1, which was based on the length of the gene and the reads count mapped to this gene [72]. The DESeq R package was used to distinguish the differential expression between the transgenic lines and WT [73]. *P*-values were adjusted using the Benjamini and Hochberg Method to assess the false results of significantly differential expression (adjusted *P*<0.01) [74]. The GOseq R package was used to analyze gene ontology (GO) enrichment of differentially expressed genes (DEGs) [75]. The DEGs were corrected at *P* < 0.05 to build GO terms. KOBAS software was used to test the statistical enrichment of differential expression in the KEGG pathway [76]. The raw data of RNA-seq were uploaded in the Sequence Read Archive database at the National Center for Biotechnology Information under access number.

### Extraction and determination of JA

To measure the JA content of each genotype, a previous method was employed with slight modification [77]. In brief, 0.5 g of two-week-old leaf tissue (growing on nutrient soil) of each genotype was ground into powder using liquid nitrogen. The powders were added with internal standards (2H5-JA 95 pmol), extracted with methanol, and incubated at– 20°C overnight. The mixture was centrifuged at 4°C for 15 min at 20,000 g. The supernatant was maintained, dried under nitrogen gas, and dissolved in 1 mL of ammonia solution (5%). The Oasis MAX SPE column was used to purify the crude extract. The purified eluent through the column was dried with nitrogen gas and finally dissolved in 200 μL of water/methanol (20:80, v/v) for UPLC-MS/MS analysis (Waters, Milford, MA, USA). The parameters of UPLC-MS/MS referred to a previously described method [77, 78]. These results were repeated more than three times.

## Results

### Construction of *Hrip1* overexpressing transgenic plants

**Fig 1:**
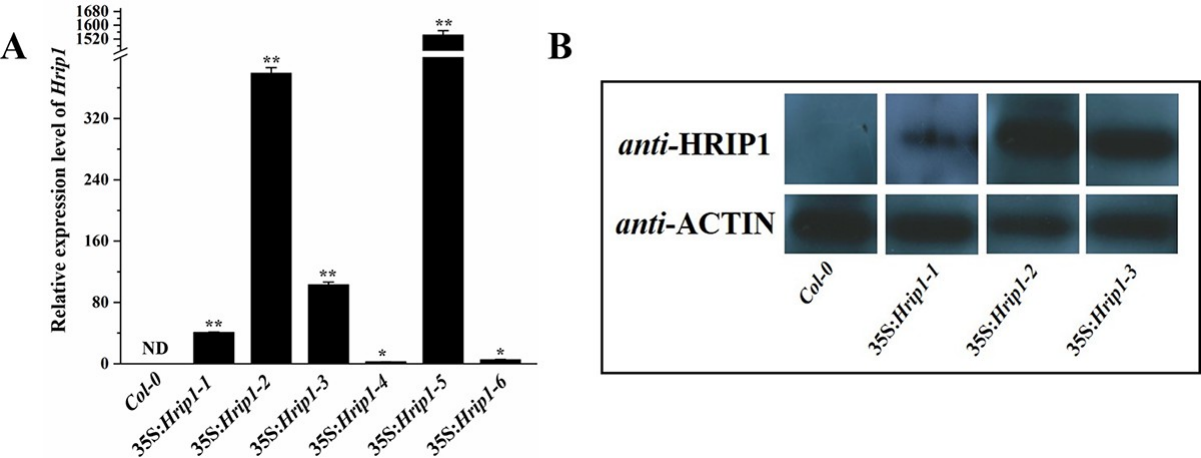
Analysis of *Hrip1* expression in transgenic plants. The *Hrip1* RNA and protein expression levels in wild-type (WT) and transgenic *Arabidopsis* were quantified through (A) real-time PCR and (B) Western blot analysis. 35S:*Hrip1-1*, 35S:*Hrip1-2*, 35S:*Hrip1-3*, 35S:*Hrip1-4*, 35S:*Hrip1-5* and 35S:*Hrip1-6*: transgenic *Arabidopsis* lines overexpressing *Hirp1*; *Col-0*: WT *Arabidopsis* lines. *Actin2* and *Actin7* were used as internal controls in real-time PCR analysis. *Actin2* was used as the internal control in Western blot analysis. Each experiment was repeated more than thrice, and similar results were obtained per run. Data are presented as the means ± SD of three independent experiments. Asterisks indicate significant differences between transgenic and WT *Arabidopsis* (Student’s *t-*test: *P < 0.05; **P < 0.01).

The gene sequence of *Hrip1* without a signal peptide was cloned and fused into the *Nco* I and *BstE* II sites of the *pCAMBIA*1301 vector with the CaMV35s promoter and transferred into *Col-0*. More than 20 positive transgenic seedlings of (T0) were screened as hygromycin-resistant. Six of the positive transgenic seeds (T1) were hygromycin-resistant and showed phenotypic ratios that corresponded to the Mendelian ratio of 3:1. All homozygote seeds of the T2 and T3 were selected and confirmed using hygromycin resistance. We checked the expression level of *Hrip1* in the independent T3 homozygote seedlings (35S:*Hrip1-1* to 35S:*Hrip1-6*) with qRT-PCR. The results showed that *Hrip1* was successfully overexpressed in *Col-0* (Fig 1A). Subsequently, we detected the Hrip1 protein expression level in three individual transgenic lines (35S:*Hrip1-1*, 35S:*Hrip1-2*, and 35S:*Hrip1-3*) using the specific antibody of Hrip1 (Anti-Hrip1). As shown in Fig 1B, the protein Hrip1 was highly expressed in *Col-0*, and the expression pattern was in accordance with the results of the *Hrip1* RNA expression pattern in qRT-PCR. To eliminate the phenotypic characteristic that was caused by transplant, three independent *Hrip1*-overexpression lines (35S:*Hrip1-1*, 35S:*Hrip1-2*, and 35S:*Hrip1-3*) with High, middle and low expression levels were selected for further analysis.

### Hrip1 accelerated bolting in *Arabidopsis* under long day treatment

**Fig 2:**
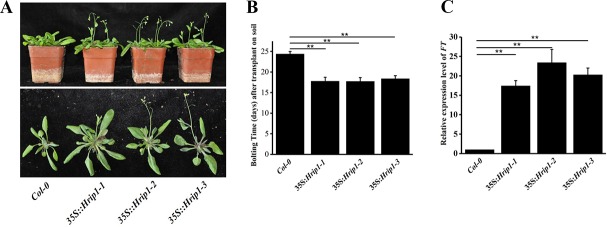
Effects of Hrip1 overexpression on the bolting time of transgenic *Arabidopsis*. (A) Bolting phenotype, (B) bolting time; bars represent standard deviation (n ≥ 30) and (C) *FT* expression levels in wild-type (WT) and *Hrip1*-overexpressing transgenic lines quantified through qRT-PCR analysis. *Actin2* and *Actin7* were used as internal controls in real-time PCR analysis. All experiments were repeated more than thrice. Data are presented as the means ± SD of three independent experiments. Asterisks indicate significant differences between transgenic and WT *Arabidopsis* lines (Student’s *t*-test: *P < 0.05; **P < 0.01).

The lines were transferred to soil to grow after all genotype seeds were germinated on MS medium for 7 days under long day conditions (16 hrs light/8 hrs night). The timing of floral induction was determined by counting the number of rosette leaves at the time of bolting (S2 Fig). The *Hrip1* transgenic plants initiated bolting at approximately 18 days after they were transplanted on nutrition soil, which was ahead of the flower bolting time compared with WT lines (approximately 24 days after growing on nutrient soil) (Fig 2A and 2B). The *Hrip1* transgenic plants were ahead by about 6 days to bolting compared with control WT.

FLOWERING LOCUS T (FT) is a mobile protein translated in leaves, and it interacts with the bZIP transcription factor FD in shoot apical meristem (SAM), and resulted in the activation of the floral meristem genes [79–81]. To explain why the bolting time of the *Hrip1* transgenic line was shorter than that of WT, the expression level of *FT* was examined via qRT-PCR using the special primers listed in S5 Table. The results showed that the expression level of *FT* was significantly upregulated in the leaves of the *Hrip1* transgenic lines compared with that in control WT prior to bolting in plants (Fig 2C).

### *Hrip1* overexpression in *Arabidopsis* enhanced the resistance to *S*.*exigua*

**Fig 3:**
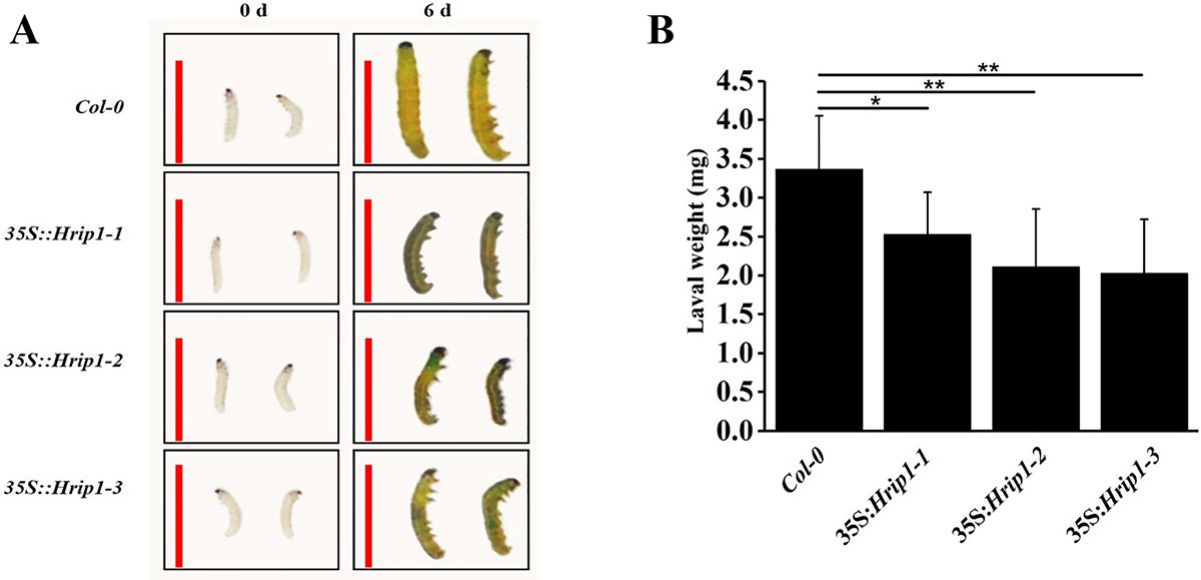
Suppressive effects of transgenic plants on the growth of *Spodoptera exigua*. (A) *S*. *exigua* individuals fed with 2-week-old transgenic plants (growing on nutrient soil) for 6 days. (B) Weight of *S*. *exigua* individuals fed with transgenic plants for 6 days. The red vertical line is shown for scale and represents 1 cm. Bars represent standard deviation (n≥30). Each experiment was repeated more than thrice. Data are presented as the means ± SD of three independent experiments. Asterisks indicate significant differences between transgenic and WT *Arabidopsis* lines (Student’s *t-*test: *P < 0.05; **P < 0.01).

Hrip1 protein infiltrated in tobacco leaves and induced SAR against tobacco mosaic virus (TMV) [67], and stimulated oxidative burst and the H_2_O_2_ accumulation at early time of application [70]. As show in S1 Fig, the H_2_O_2_ level in leaves of *Hrip1*-overexpression transgenic plants and WT was not significantly different. Furthermore, Hrip1 can elevate the expression level of *LOX2*, which is a JA synthesis-related gene, after Hrip1 is infiltrated in tobacco leaves; therefore, we speculated that Hrip1 may be resistant to pests [67, 82]. The second instar larvae of *S*.*exigua* were fed with rosette leaves (after the plants grew for approximately two weeks on nutrient soil) for 6 days, and the leaves were replaced every 2 days. After growth for 6 days, the weight of larvae fed with leaves of the *35S*:*Hrip1-1*, *35S*:*Hrip1-2*, and *35S*:*Hrip1-3* transgenic lines significantly decreased by 27.30%, 37.39%, and 39.76% compared with the weight of larvae fed with leaves of WT (Fig 3A and 3B). Taken together, *Hrip1* could enhance resistance to *S*.*exigua* in *Arabidopsis*.

### Analysis of RNA-sequencing results

**Fig 4:**
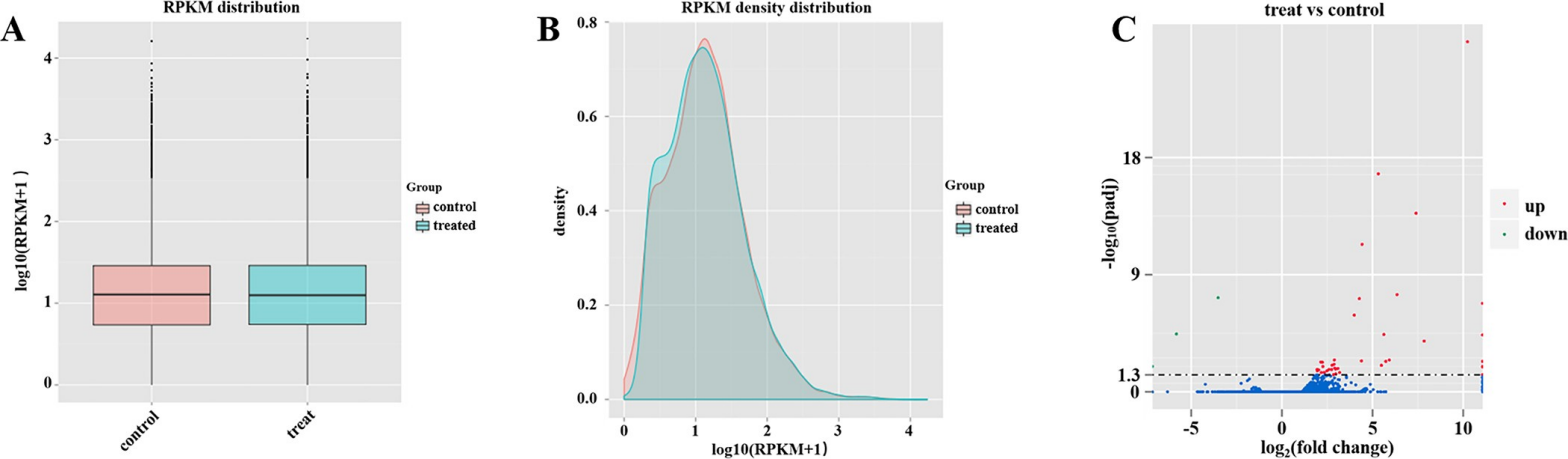
Comparison of expression levels in WT (control, AtRNA_C) and transgenic (treat, AtRAN_H) *Arabidopsis*. (A) Comparison of RPKM distribution between WT and transgenic *Arabidopsis*. The y-axis indicates the log_10_(RPKM+1) values of genes. The x-axis indicates samples: WT (control) and transgenic (treat) *Arabidopsis* lines. (B) Summary of the RPKM density distribution of WT and transgenic *Arabidopsis* lines. The y-axis indicates density values. The x-axis indicates the log_10_(RPKM+1) values of genes. Red and blue represent WT and transgenic *Arabidopsis* lines, respectively. (C) Volcano plot of differentially expressed genes. The y-axis indicates *−*log_10_(padj) values with significant differences. The x-axis indicates the log_2_(fold-change) values. Blue points represent genes that are not considerably differentially expressed, red points represent considerably upregulated genes, and green points represent considerably downregulated genes.

Three biological replicates of WT (C1, C2, and C3) and transgenic plants 35:*Hrip1-2* (H1, H2, and H3) were used to perform RNA sequencing, which was completed with an Illumina HiSeq 2000 platform (Novogene Bioinformatics Technology Co., Ltd, Beijing, China). To assess the quality of the sequencing data, we used the error rate and base contents as reference (S2 Table). The cleaned sequences of samples were mapped to the *Arabidopsis* reference genome analyzed with software TopHat v2.0.9 [83, 84], and the results of special reads matched to the genome are showed in S3 Table. To evaluate the expression level of each gene on the basis of RPKM [72], we set the RPKM > 1 as threshold for significant gene expression. More than half of the matched genes were significantly expressed (S3 Table). We calculated the average RPKM value from the three biological replicates of each gene and then compared the difference in gene expression levels between WT and transgenic plants 35:*Hrip1-2* (Fig 4A and 4B). To explore the gene expression difference between the transgenic lines 35:*Hrip1-2* (treat, AtRNA_H) and WT (control, AtRNA_C), the average read count data were used for analysis. The fold change of DEGs was determined based on the ratio of read count value of one gene between AtRNA_H and AtRNA_C. We set p_adj_ < 0.01 as the analysis condition to statistically decide the DEGs AtRNA_H and AtRNA_C. Subsequently, we found 40 genes that were upregulated and three genes that were significantly downregulated in AtRNA_H (Fig 4C).

**Fig 5:**
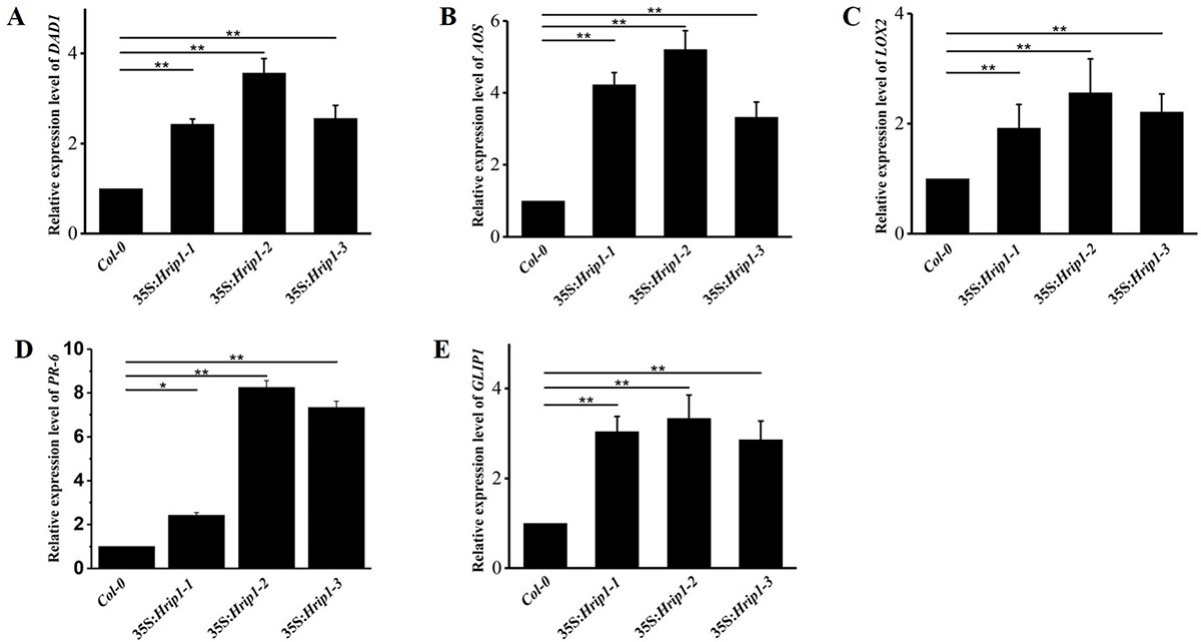
Verification of differentially expressed DGE results using qRT–PCR. (A–E) The relative expression level of each gene significantly fold-changed in transgenic *Hrip1*-overexpressing *Arabidopsis* lines. *Actin2* and *Actin7* were used as internal controls in real-time PCR analysis. Each experiment was repeated more than thrice. Data are presented as the means ± SD of three independent experiments. Asterisks indicate significant differences between transgenic and WT *Arabidopsis* (Student’s *t-*test: **P < 0.01).

Six DEGs between AtRNA_H and AtRNA_C were used for further analysis. Flowering locus (FT), a mobile protein in the leaf that determines the flower bolting time, showed 2.9-fold changes in transgenic line 35:*Hrip1-2* (S4 Table). Some genes were involved in JA biosynthesis, such as defective anther dehiscence 1 (*DAD1*), *LOX2*, and *AOS*, and these genes were upregulated 1.9-, 2.6- and 2.3-fold, respectively (S4 Table). DEGs results revealed that *GLIP1*, clade IIIa of GDSL-type esterases/lipases (*AtGELP*), which participates in the activation of biotic responses, exhibited 2.2-fold changes. PR6, a low-molecular-weight peptide grouped into PR protein, was only expressed in the transgenic line of *Hrip1*-overexpression (S4 Table). To confirm the DEGs results, we collected leaves from the transgenic lines (*35S*:*Hrip1-1*, *35S*:*Hrip1-2*, *and 35S*:*Hrip1-3*) and WT of *Arabidopsis* plants that were not bolting (approximately two weeks after growing on nutrient soil). All of the interesting genes were confirmed with qRT-PCR with the specific primers listed in S5 Table. Changes in the expression level of genes of interest are shown in Fig 2C, and Fig 5. These findings were in agreement with the results of DEGs.

The 40 DEGs of upregulation were classified using GO enrichment to illustrate their potential functions, which were divided into 22 GO accessions with the most enrichment (P_corrected_ < 0.05; S3 Fig). These categories were as follows: “response to stimulus” (GO: 0050896), “biological regulation” (GO: 0065007), “cellular process” (GO: 0009887) and “metabolic process” (GO: 0008152). The two GO terms of molecular accessions were “catalytic activity” (GO: 0003824) and “binding” (GO: 0005488). Most DEGs were from biological process activity. The FT was presented in the top of four significant GO terms of biological process, namely, “developmental process” (GO: 0032502), “multicellular organismal process” (GO: 0032501), “reproduction” (GO: 0000003), and “reproductive process” (GO: 0022414). The JA biosynthesis genes (*DAD1*, *LOX2*, and *AOS*) and *GDSL1* were included in the GO terms “response to stimulus”, “regulation of biological process”, and “biological process”.

The DEGs were analyzed using KEGG enrichment to illustrate the signal transduction or metabolism network that these genes took part in. The DEGs were divided into 15 pathways. The JA biosynthesis genes *DAD1*, *LOX2*, and *AOS* were involved in the “α-linolenic acid metabolism” (ath00592) pathway, which was significantly enriched at P_corrected_ < 0.01 (S4 Fig). The protein FT mentioned above was involved in the pathway named “circadian rhythm-plant”, identified at P_corrected_ = 0.2146 (S4 Fig); this protein acted as a long-distance signal to induce flowering [85].

### JA content were slightly higher in transgenic lines than in WT

**Fig 6:**
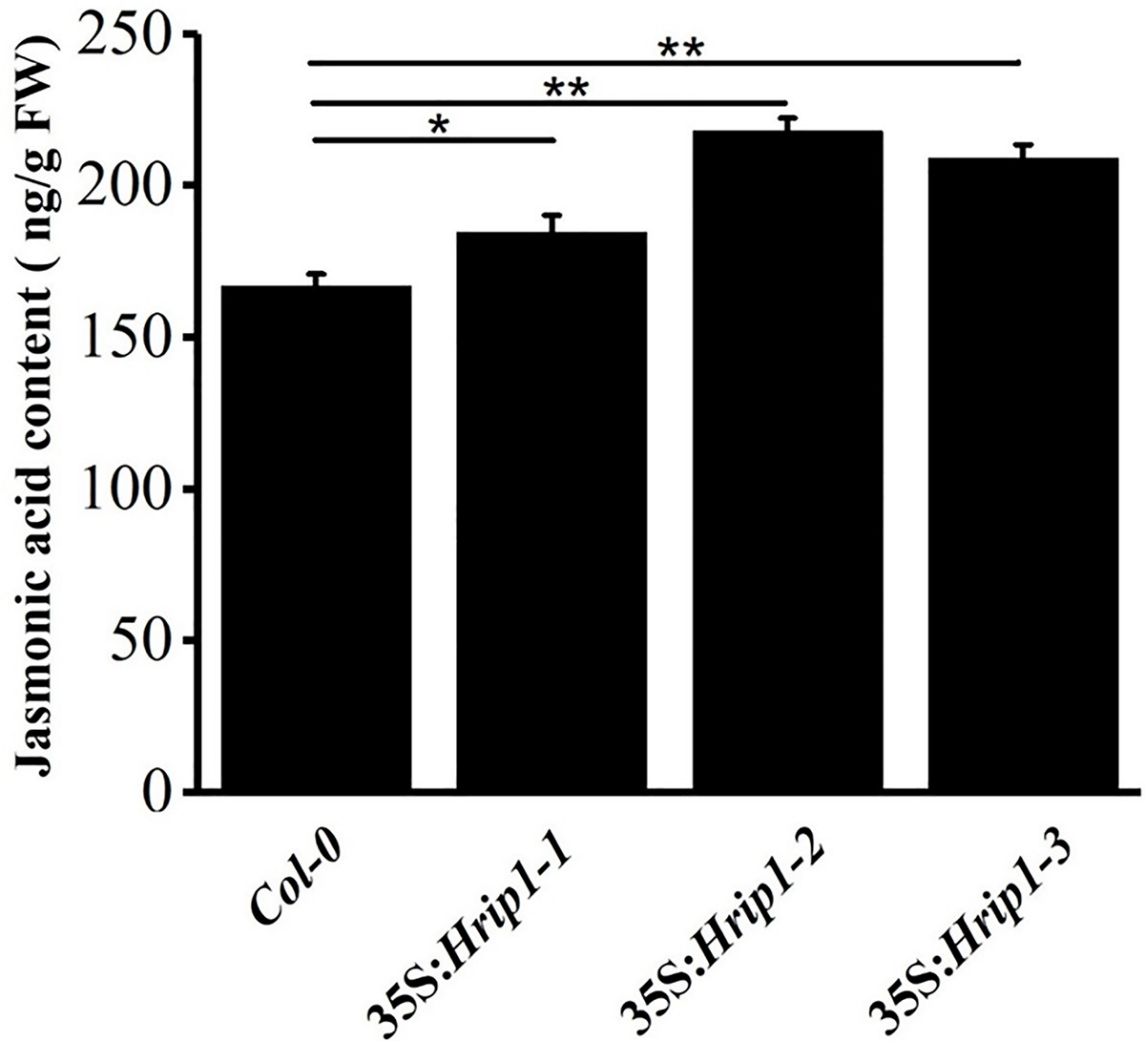
Jasmonic acid contents of rosette leaves from wild-type (WT) and *Hrip1-*overexpressing transgenic lines. Jasmonic acid contents of 2-week-old rosette leaves from WT, 35S:*Hrip1-1*, 35S:*Hrip1-2* and 35S:*Hrip1-3* transgenic plants (growing on nutrient soil). Data are presented as the means ± SE of three biological replicates. Asterisks represent a significant difference between transgenic and WT *Arabidopsis* lines (Student’s *t*-test *P < 0.05, **P < 0.01).

Some genes involved in JA biosynthesis were upregulated in transgenic lines of *Hrip1*-overexpression, so we speculated that JA level must be enhanced in transgenic lines. To verify this hypothesis, JA was isolated from leaves (approximately two weeks after plants grew on nutrient soil) and quantified via UPLC-MS/MS. In the transgenic line of *Hrip1*-overexpression, the contents of endogenous JA were examined to compared with WT *Arabidopsis*, and values of 184.70, 218.21, and 209.31 ng.g-1 fresh weight were obtained (Fig 6). In the transgenic lines of *Hrip1*-overexpression, the expression level of genes involved in JA biosynthesis were enhanced and the JA level increased. Therefore, Hrip1 could trigger JA biosynthesis.

## Discussion

The novel elicitor Hrip1 was purified from the necrotrophic fungus *A*. *tenuissima* using an ion exchange chromatography column [67]. In our previous work, Hrip1 was found to induce SAR in tobacco and enhance the resistance of plants to salt and drought [56, 67]. Here, we showed that Hrip1 promoted flower bolting and resistance to insects in *Arabidopsis*.

The previously article reported that the transgenic plants can enhance biotic and abiotic resistance when Hrip1 is overexpressed in *Arabidopsis* [56]. The *Hrip1* transgenic lines of *Arabidopsis* displayed significantly higher changes in plant height, silique length, and plant dry weight, and the *Hrip1* gene was induced by the rd29A promoter [56]. In this assay, we found that the transgenic plants of *Hrip1*-overexpression had other biological functions, including resistance to insects (Fig 3) and early bolting time in transgenic plants (Fig 2). These results were similarly observed in other elicitors isolated from different pathogens. The Harpin protein is an elicitor with many functions, including increased resistance to fungi and insects in many plants; it also regulates plant growth and flower bolting time by influencing the expression of plants, such as JA signaling-related genes and components of the ethylene (ET) signaling pathway [59–62, 86–90]. PevD1, an elicitor isolated from the cotton *Verticillium* wilt fungus *V*. *dahliae*, which can trigger resistance to pathogens in many plants by affecting the plant signaling pathway (including phytohormones JA, calcium ions, and WRKY) and regulating plant growth [64–66]. The enzymes of JA biosynthesis, PR-6 and GLIP1, were activated to enhance plant resistance to insects in *Hrip1*-overexpressing *Arabidopsis*.

The flower bolting time was determined by day length and mobile floral stimulus in leaves [35]. FT is a member of the phosphatidylethanolamine binding proteins, which is a key flowering promoter [80, 81, 91, 92] that is triggered by CONSTANTS (CO) in vascular tissues of leaves under long days [81, 93–96]. The FT protein moves from leaves to the SAM through the phloem to induce flowering in *Arabidopsis* [85, 97–99]. Furthermore, FT interacts with FD in SAM, resulting in the activation of SUPPRESSOR OF OVEREXPRESSION OF CONSTANS 1 (SOC1) and APETALA1 to initiate flower development [80, 81]. Our DEG results revealed that the transcription level of FT was significantly higher than that of *Col-0* (S4 Table), which was confirmed by qRT-PCR (Fig 2C). Other elicitors, such as PevD1 and Harpin Hpa1, were reported to contribute to flower development among the plants [90, 100]. These results suggested that the expression of *FT* may be affected directly or indirectly in the transgenic lines of *Hrip1-*overexpression.

The phytohormone JA plays an important role to triggering plant responses against biotic infections, including insect and pathogen [17, 101–103]. Genes responsive to JA were rapidly triggered by JA biosynthesized by a series of well-organized enzymes, including DAD1, LOX, AOS, AOC, and OPDA [15, 17, 32]. In our assay, the results of the DEGs and qRT-PCR demonstrated that three genes of JA-biosynthesis, namely, *DAD1*, *LOX-2*, and *AOS*, were highly expressed in the transgenic lines of *Hrip1-*overexpressing compared with WT (Fig 4 and S4 Table). The endogenous JA levels in transgenic lines of *Hrip1-*overexpression detected by LC-MS/MS were higher than those in the control *Col-0* in *Arabidopsis* (Fig 6). These results proved that some genes involved in JA biosynthesis were activated when Hrip1 was overexpressed in *Arabidopsis*, which may laterally explain the transgenic resistance to larvae of *S*.*exigua* (Fig 3) and necrotrophic fungi [56, 67]. Similarly, the Harpin protein was reported to triggered the expression of *TDF89H1* and *TDF249H2* in *Phalaenopsis orchids*; these genes were similar to *JAR1* and *JAR4* of *Arabidopsis* [62]. In the results of DEGs, no significantly expressed gene related to the SA signaling pathway, such as *PR1*, was found. However, a pathogenesis-related peptide *PR-6* was highly expressed in the transgenic lines of *Hrip1*-overexpression, which played a major role in defense action [42–44, 46].

In *Arabidopsis*, 105 GDSL-type esterase/lipase genes were divided into four clades based on function, namely, morphological development, abiotic stress response, secondary metabolism, and pathogen defense [49]. *GLIP1* belongs to clade IIIa of *AtGELP*; it participates in the plant’s immunity action via the ethylene signaling pathway and enhances the expression of *ERF1* and suppresses the expression of *EIN3* [51, 52]. Furthermore, *SID2*, a SA biosynthesis gene, is significantly expressed compared with WT, when *GLIP1* is overexpressed in *Arabidopsis*. In the transgenic lines of *Hrip1*-overexpression, *GLIP1* and *GLIP4* were differentially expressed compared with WT (S4 Table), and these results were confirmed by qRT-PCR (Fig 5). These results implied that *GLIP* could be activated when *Hrip1* was overexpressed in *Arabidopsis*, which may induce the immune system of the plant via the ethylene signaling pathway to lead to fungal resistance [56, 63].

## Additional information

Sequence data are available in the Sequence Read Archive (SRA) database at the National Center for Biotechnology Information (NCBI) with access number PRJNA498541.

## Supporting information

S1 Fig H_2_O_2_ accumulation in *Arabidopsis* leaves of *Hrip1*-overexpression transgenic plants and WT. Accumulation of H_2_O_2_ in leaves of transgenic plants of *Hrip1*-overexpression and WT which grew on MS medium for 7days and 14 days. Compared with WT, *Hrip1*-overexrpession plants are shown to accumulate same levels of H_2_O_2_.
(DOCX)

S2 Fig Rosette leaves number of wild type and transgenic lines of *Hrip1*-overexpression. Timing of floral initiation in transgenic *Arabidopsis* plants was determined by counting the number of rosette leaves formed at the time of bolting (mean ± SE, n = 30 plants per treatment), each experiment was repeated more than thrice. Asterisks indicate significant differences between transgenic and WT *Arabidopsis* (Student’s *t*-test: **P < 0.01).
(DOCX)

S3 Fig Gene Ontology (GO) classification of the DEGs. The unigenes were classified in three main categories: biological process, cellular location, and molecular function.
(DOCX)

S4 Fig Scattered Plot of KEGG pathway terms of differentially expressed genes. Dot size represents the number of different genes and rich factor indicates the value of pcorrected.
(DOCX)

S1 Table The quality of sequencing data. 1) Sample name: the names of samples.
2) Raw reads: the original sequencing reads counts.
3) Clean reads: number of reads after filtering.
4) Clean base: clean reads number multiply read length, saved in G unit.
5) Error rate: average sequencing error rate, which is calculated by Qphred = -10log10(e).
6) Q20: percentages of bases whose correct base recognition rates are greater than 99% in total bases.
7) Q30: percentages of bases whose correct base recognition rates are greater than 99.9% in total bases.
8) GC content: percentage of G and C in total bases.
(DOCX)

S2 Table Overview of mapping status. 1) Total number of filtered reads (Clean data).
2) Total number of reads that can be mapped to the reference genome. In general, this number should be larger than 70% when there is no contamination and the correct reference genome is chosen.
3) Number of reads that can be mapped to multiple sites in the reference genome. This number is usually less than 10% of the total.
4) Number of reads that can be uniquely mapped to the reference genome.
5) Number of reads that map to the positive strand (+) or the minus strand (-).
6) Splice reads can be segmented and mapped to two exons (also named junction reads), whereas non-splice reads can be mapped entirely to a single exon. The ratio of splice reads depends on the insert size used in the RNA-seq experiments.
(DOCX)

S3 Table The number of genes with different expression levels. (DOCX)

S4 Table List of differential genes between AtHrip1 and AtWT. (DOCX)

S5 Table Primer sequence designed for this study. (DOCX)
