# Supplementary figures and images for: The protein elicitor Hrip1 enhances resistance to insects and early bolting and flowering in Arabidopsis thaliana
Source: PLoS One. 2019 Apr 25;14(4):e0216082. doi: 10.1371/journal.pone.0216082 (PMC6483360; doi:10.1371/journal.pone.0216082)

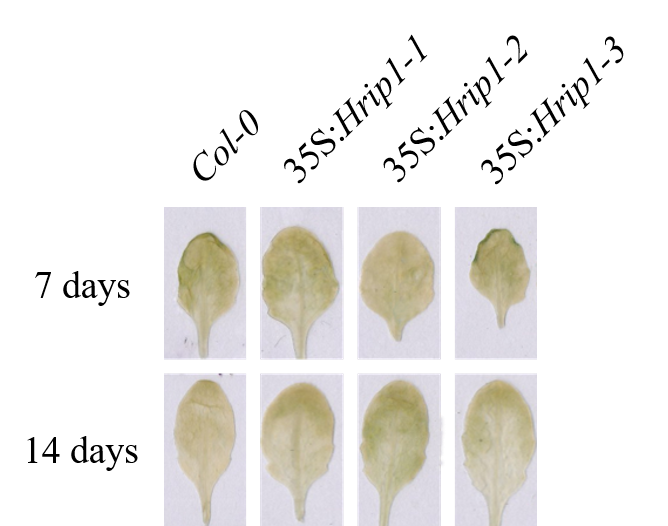

Supplement: S1 Fig — Accumulation of H2O2 in leaves of transgenic plants of Hrip1-overexpression and WT which grew on MS medium for 7days and 14 days. Compared with WT, Hrip1-overexrpession plants are shown to accumulate same levels of H2O2. (DOCX) [file pone.0216082.s001.docx]

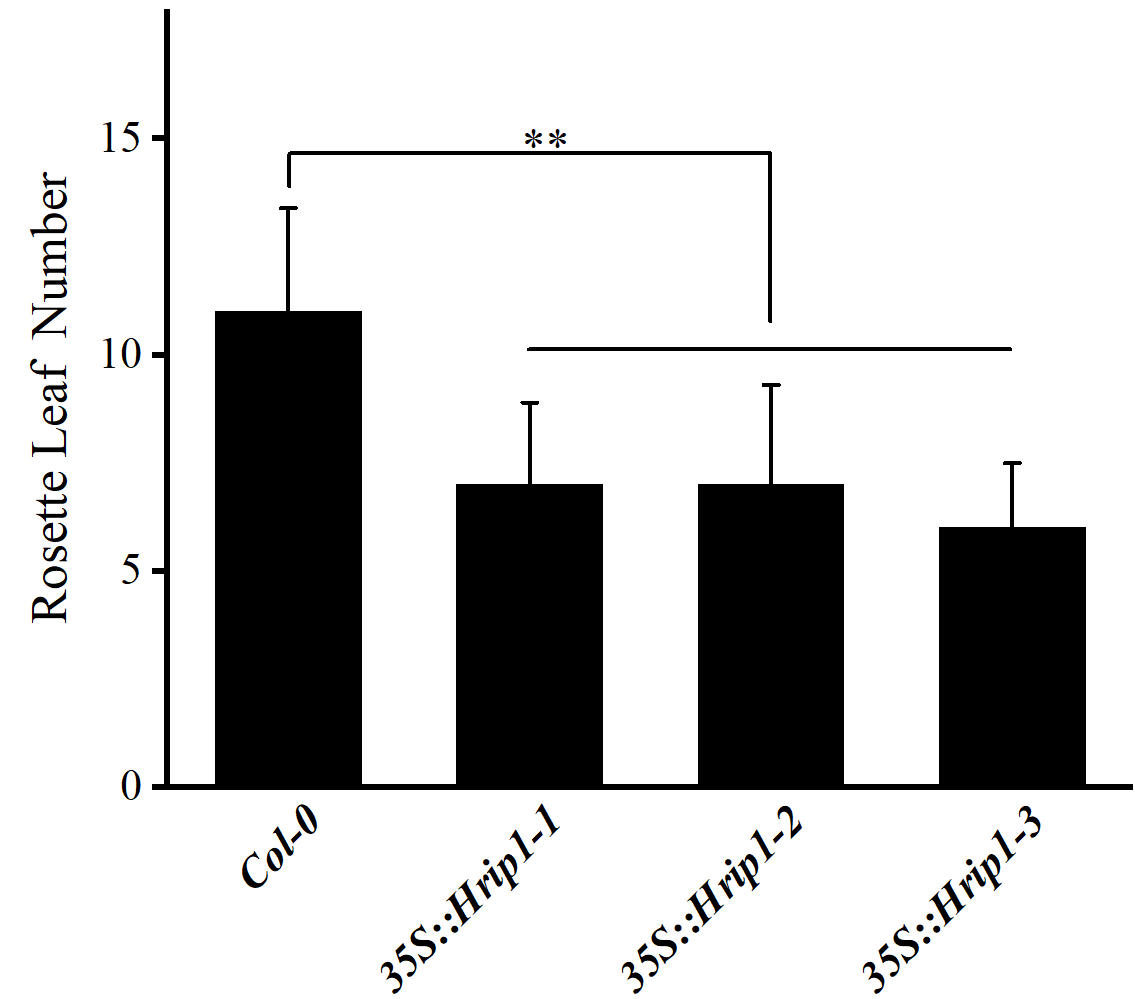

Supplement: S2 Fig — Timing of floral initiation in transgenic Arabidopsis plants was determined by counting the number of rosette leaves formed at the time of bolting (mean ± SE, n = 30 plants per treatment), each experiment was repeated more than thrice. Asterisks indicate significant differences between transgenic and WT Arabidopsis (Student’s t-test: **P < 0.01). (DOCX) [file pone.0216082.s002.docx]

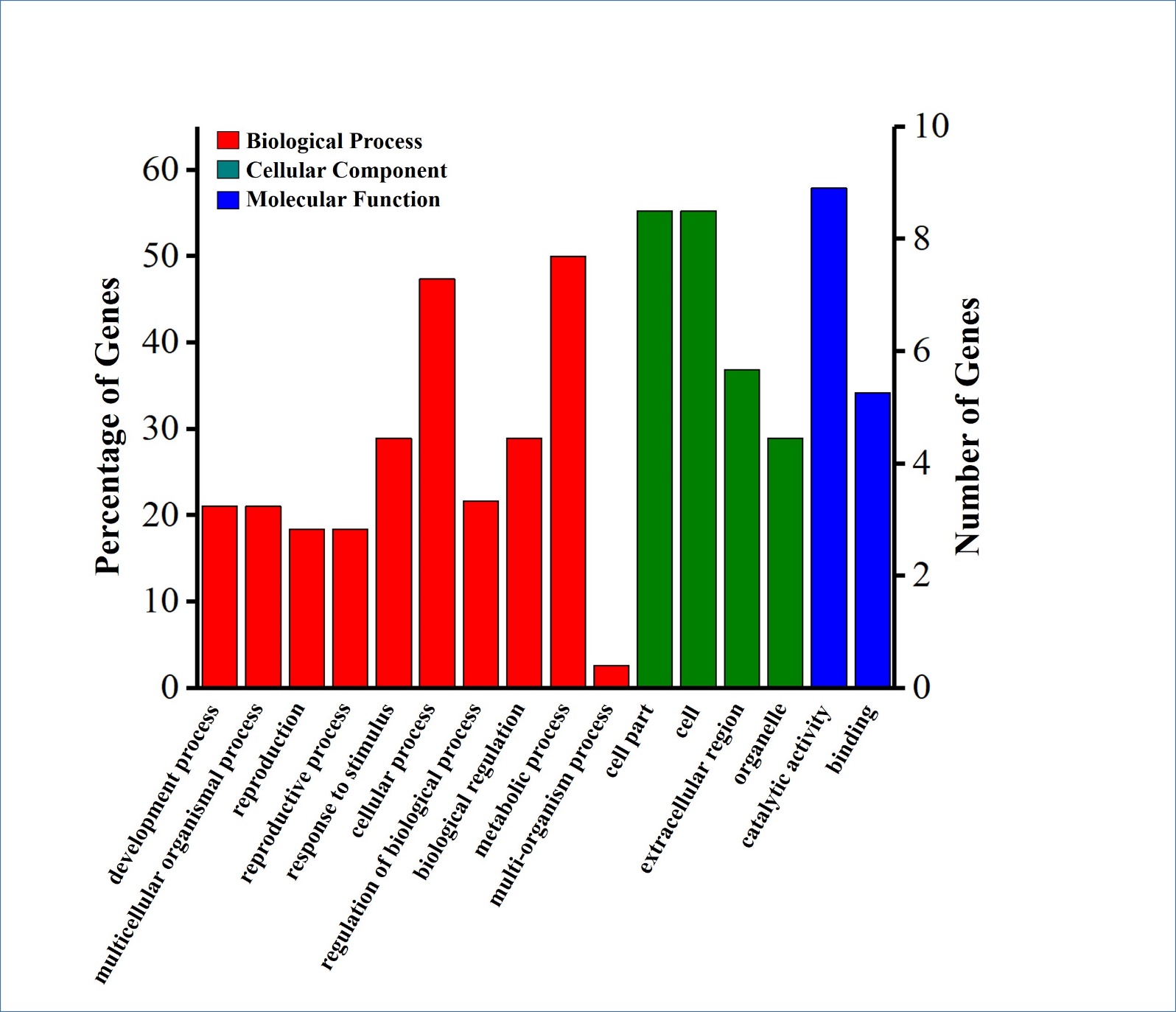

Supplement: S3 Fig — The unigenes were classified in three main categories: biological process, cellular location, and molecular function. (DOCX) [file pone.0216082.s003.docx]

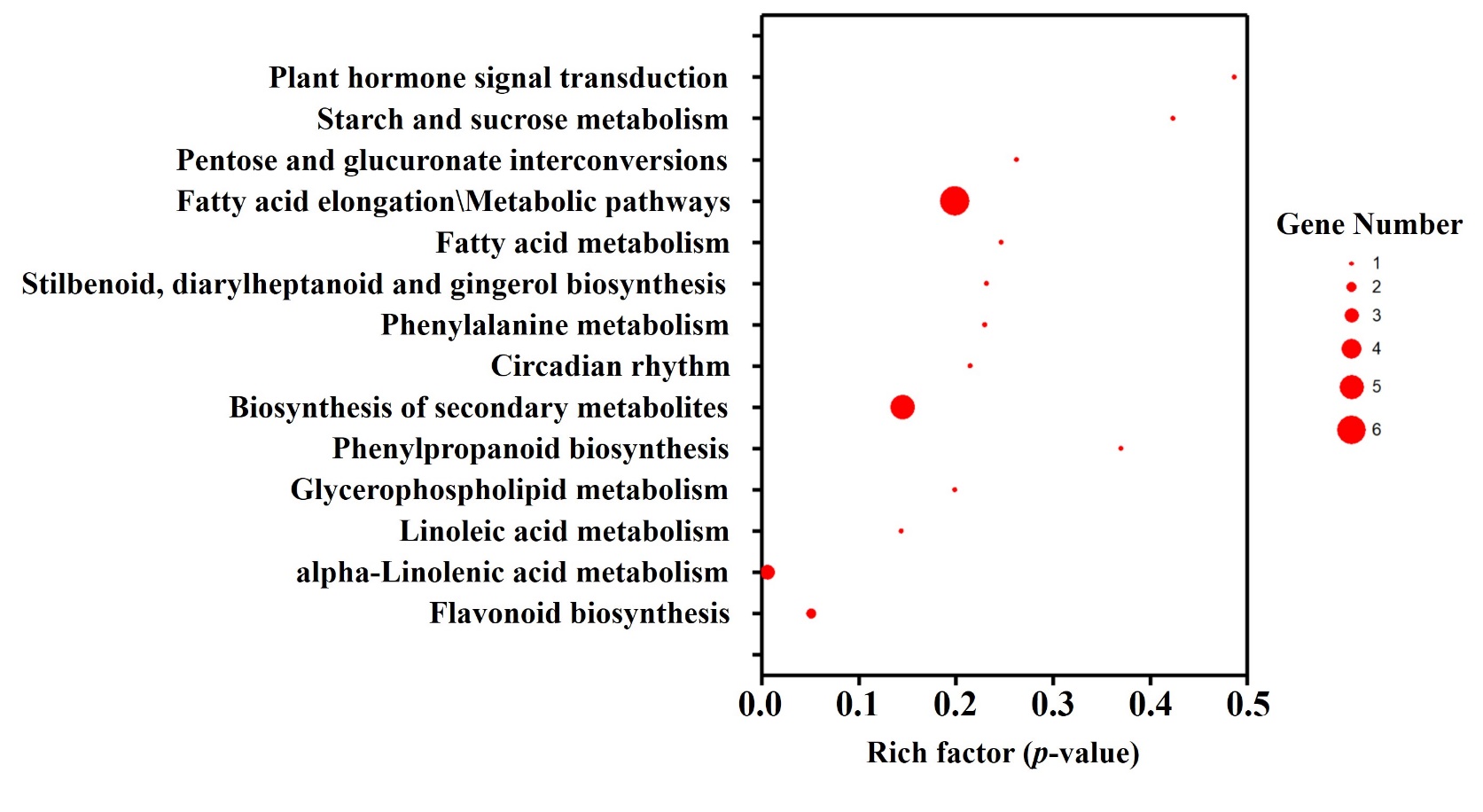

Supplement: S4 Fig — Dot size represents the number of different genes and rich factor indicates the value of pcorrected. (DOCX) [file pone.0216082.s004.docx]
