# Supplementary material for: The protein elicitor Hrip1 enhances resistance to insects and early bolting and flowering in Arabidopsis thaliana
Source: PLoS One. 2019 Apr 25;14(4):e0216082. doi: 10.1371/journal.pone.0216082 (PMC6483360; doi:10.1371/journal.pone.0216082)
Supplement: S1 Table — 1) Sample name: the names of samples. 2) Raw reads: the original sequencing reads counts. 3) Clean reads: number of reads after filtering. 4) Clean base: clean reads number multiply read length, saved in G unit. 5) Error rate: average sequencing error rate, which is calculated by Qphred = -10log10(e). 6) Q20: percentages of bases whose correct base recognition rates are greater than 99% in total bases. 7) Q30: percentages of bases whose correct base recognition rates are greater than 99.9% in total bases. 8) GC content: percentage of G and C in total bases. (DOCX) [file pone.0216082.s005.docx]

| Sample  name | Raw  reads | | Clean  reads | Clean  bases | Error  Rate (%) | Q20 (%) | Q30 (%) | GC  Content (%) |
| --- | --- | --- | --- | --- | --- | --- | --- | --- |
| C1 | 13274242 | 13244570 | | 0.99G | 0.03 | 94.58 | 87.77 | 45.06 |
| C2  C3  H1 | 13179929  13283567  15120486 | 13175164  13256238  15113614 | | 0.99G  0.99G  1.14G | 0.03  0.03  0.03 | 94.71  94.36  94.83 | 88.01  88.65  88.18 | 45.36  45.21  45.17 |
| H2 | 15229054 | 15194156 | | 1.13G | 0.03 | 94.14 | 87.11 | 45.70 |
| H3 | 15123461 | 15116347 | | 1.14G | 0.03 | 94.87 | 88.36 | 45.43 |
