# Supplementary material for: The protein elicitor Hrip1 enhances resistance to insects and early bolting and flowering in Arabidopsis thaliana
Source: PLoS One. 2019 Apr 25;14(4):e0216082. doi: 10.1371/journal.pone.0216082 (PMC6483360; doi:10.1371/journal.pone.0216082)
Supplement: S2 Table — 1) Total number of filtered reads (Clean data). 2) Total number of reads that can be mapped to the reference genome. In general, this number should be larger than 70% when there is no contamination and the correct reference genome is chosen. 3) Number of reads that can be mapped to multiple sites in the reference genome. This number is usually less than 10% of the total. 4) Number of reads that can be uniquely mapped to the reference genome. 5) Number of reads that map to the positive strand (+) or the minus strand (-). 6) Splice reads can be segmented and mapped to two exons (also named junction reads), whereas non-splice reads can be mapped entirely to a single exon. The ratio of splice reads depends on the insert size used in the RNA-seq experiments. (DOCX) [file pone.0216082.s006.docx]

| Sample name | C1 | C2 | C3 | H1 | H2 | H3 |
| --- | --- | --- | --- | --- | --- | --- |
| Total reads  Total mapped | 13244570  11770905 (88.87%) | 13175164  11856862 (89.99%) | 13256238  11763451(88.65%) | 15113614  13330331 (88.2%) | 15194156  13645968 (89.81%) | 15116347  13340257(88.36%) |
| Multiple mapped | 186648 (1.41%) | 194362 (1.48%) | 185783 (1.36%) | 220528 (1.46%) | 198929 (1.31%) | 2236721 (1.53%) |
| Uniquely mapped | 11584257 (87.46%) | 11662500 (88.52%) | 11578325(86.73%) | 13109803 (86.74%) | 13447039 (88.5%) | 13236739 (87.25%) |
| Reads map to “+” | 5794812 (43.75%) | 5829807 (44.25%) | 5775921 (43.14%) | 6545711 (43.31%) | 6721025 (44.23%) | 6551783 (43.83%) |
| Reads map to “-”  Non-splice reads  Splice reads | 5789445 (43.71%)  8825380 (66.63%)  2758877 (20.83%) | 5832693 (44.27%)  8891275 (67.49%)  2771225 (21.03%) | 5773592 (43.24%)  8815632 (65.72%)  2748593 (19.79%) | 6564092 (43.43%)  10061250 (66.57%)  3048553 (20.17%) | 6726014 (44.27%)  10276278 (67.63%)  3170761 (20.87%) | 65723584 (44.07%)  10184375 (67.26%)  3063637 (20.57%) |
