# Supplementary material for: The protein elicitor Hrip1 enhances resistance to insects and early bolting and flowering in Arabidopsis thaliana
Source: PLoS One. 2019 Apr 25;14(4):e0216082. doi: 10.1371/journal.pone.0216082 (PMC6483360; doi:10.1371/journal.pone.0216082)
Supplement: S3 Table — (DOCX) [file pone.0216082.s007.docx]

| RPKM Interval | C1 | C2 | C3 | H1 | H2 | H3 |  |
| --- | --- | --- | --- | --- | --- | --- | --- |
| 0~1 | 14952(44.50%) | 14997(44.63%) | 14981(44.57%) | 14801(44.05%) | 14390(42.83%) | 14931(43.87%) |  |
| 1~3 | 2789(8.30%) | 2777(8.26%) | 2768(8.21%) | 3037(9.04%) | 3027(9.01%) | 3042(9.11%) |  |
| 3~15 | 7658(22.79%) | 7647(22.76%) | 7665(22.83%) | 7860(23.39%) | 7828(23.30%) | 7833(23.31%) |  |
| 15~60 | 6017(17.91%) | 6004(17.87%) | 6031(18.05 %) | 5687(16.93%) | 6041(17.98%) | 6056(17.84%) |  |
| >60 | 2185(6.50%) | 2176(6.48%) | 2196(6.57%) | 2216(6.60%) | 2315(6.89%) | 2316(6.74%) |  |
