# Supplementary material for: The protein elicitor Hrip1 enhances resistance to insects and early bolting and flowering in Arabidopsis thaliana
Source: PLoS One. 2019 Apr 25;14(4):e0216082. doi: 10.1371/journal.pone.0216082 (PMC6483360; doi:10.1371/journal.pone.0216082)
Supplement: S4 Table — (DOCX) [file pone.0216082.s008.docx]

| No. | Gene_ID | Readcount (AtRNA_H) | Readcount  (AtRNA_C) | log2 Fold Change | p_adj_ | Annotation |
| --- | --- | --- | --- | --- | --- | --- |
| 1 | AT3G44042 | 654.0957 | 0.5404 | 10.241 | 1.25E-27 | SADHU3-1. Member of Sadhu non-coding retrotransposon family. In some natural accessions the allele is methylated and silenced. |
| 2 | AT2G46960 | 124.5052 | 0.5404 | 7.8481 | 0.0001 | Member of CYP709B |
| 3 | AT4G04270 | 182.2571 | 1.0807 | 7.3978 | 1.89E-14 | CACTA-like transposase family (Ptta/En/Spm), has a 9.7e-78 P-value blast match to At5g29026.1/8-244 CACTA-like transposase family (Ptta/En/Spm) (CACTA-element) |
| 4 | AT1G56100 | 86.4574 | 1.06125 | 6.3482 | 3.41E-08 | Plant invertase/pectin methylesterase inhibitor superfamily protein |
| 5 | AT1G61720 | 32.7507 | 0.5404 | 5.9215 | 0.0035 | BANYULS is a negative regulator of flavonoid biosynthesis, mutants accumulate flavonoid pigments in their seed coat, putative oxidoreductase. It is thought that a ternary complex composed of TT2, TT8 and TTG1 is necessary for correct expression of BAN in seed endothelium. |
| 6 | AT4G15750 | 57.3626 | 1.0807 | 5.73 | 0.0046 | Plant invertase/pectin methylesterase inhibitor superfamily protein |
| 7 | AT5G47350 | 71.6513 | 1.6016 | 5.4834 | 0.0093 | Alpha/beta-Hydrolases superfamily protein |
| 8 | AT4G12960 | 1351.8920 | 33.9599 | 5.315 | 1.75E-17 | Gamma interferon responsive lysosomal thiol (GILT) reductase family protein |
| 9 | AT1G67105 | 948.2196 | 44.28034 | 4.4205 | 4.65E-12 | Unknown |
| 10 | AT2G25540 | 44.0313 | 2.10302 | 4.388 | 0.0042 | Cellulose synthase 10 |
| 11 | AT3G20210 | 174.2376 | 9.0109 | 4.2732 | 6.88E-08 | AEP4 encodes a vacuolar processing enzyme with caspase-1-like activity that is specifically expressed in inner integument of developing seeds. |
| 12 | AT5G49180 | 144.8515 | 9.1083 | 3.9913 | 1.28E-06 | Encodes a putative pectin methylesterase. |
| 13 | AT5G07420 | 48.3442 | 5.3257 | 3.1823 | 0.0030 | Pectin lyase-like superfamily protein |
| 14 | AT5G26692 | 72.3498 | 8.5289 | 3.0845 | 0.0062 | Encodes a Plant thionin family protein |
| 15 | AT5G59810 | 53.8135 | 6.8689 | 2.9698 | 0.0018 | ATSBT5.4. Subtilase family protein |
| 16 | AT3G14520 | 67.0144 | 8.6069 | 2.9609 | 0.0077 | SESTERTERP-ENE SYNTHASE 1 is a sesterterpene synthase responsible for the biosynthesis of the tricyclic sesterterpene (+)-thalianatriene with a 11-6-5 fused ring system. |
| 17 | AT4G12870 | 183.8061 | 24.4087 | 2.9127 | 0.0051 | Gamma interferon responsive lysosomal thiol (GILT) reductase family protein |
| 18 | AT1G65480 | 116.1494 | 15.6705 | 2.8899 | 0.0035 | FLOWERING LOCUS T (FT) together with LFY, promotes flowering and is antagonistic with its homologous gene, TERMINAL FLOWER1 (TFL1). FT is expressed in leaves and is induced by long day treatment. Either the FT mRNA or protein is translocated to the shoot apex where it induces its own expression. Recent data suggests that FT protein acts as a long-range signal. FT is a target of CO and acts upstream of SOC1. |
| 19 | AT3G20520 | 149.2297 | 20.68463 | 2.8509 | 0.0075 | GDPDL5. Encodes a member of the glycerophosphodiester phosphodiesterase like (GDPD-like) family. |
| 20 | AT2G21490 | 93.4633 | 12.7155 | 2.8778 | 0.0078 | Dehydrin LEA |
| 21 | AT1G20130 | 365.8067 | 53.8805 | 2.7632 | 0.0090 | GDSL-motif esterase/acyltransferase/lipase. Enzyme group with broad substrate specificity that may catalyze acyltransfer or hydrolase reactions with lipid and non-lipid substrates. |
| 22 | AT4G26220 | 82.3762 | 12.3115 | 2.7422 | 0.0094 | CCOAOMT7 encodes a caffeoyl-coenzyme A O-methyltransferase (CCoAOMT)-like protein with a strong preference for methylating the para position of flavanones and dihydroflavonols, whereas flavones and flavonols are methylated in the meta-position. |
| 23 | AT3G45140 | 98.9416 | 16.3617 | 2.5963 | 0.0097 | LOX2 is chloroplast lipoxygenase required for wound-induced jasmonic acid accumulation in Arabidopsis. Mutants are resistant to Staphylococcus aureus and accumulate salicylic acid upon infection. The mRNA is cell-to-cell mobile. |
| 24 | AT5G19580 | 1141.2401 | 226.1775 | 2.3351 | 0.0027 | Glyoxal oxidase-related protein |
| 25 | AT5G42650 | 278.2559 | 58.1545 | 2.2585 | 0.0014 | AOS a member of the cytochrome p450 CYP74 gene family that functions as an allene oxide synthase. This enzyme catalyzes dehydration of the hydroperoxide to an unstable allene oxide in the JA biosynthetic pathway. It shows a dual catalytic activity, the major one being a 13-AOS but also expressing a 9-AOS activity. |
| 26 | AT5G38760 | 106.7800 | 19.1025 | 2.4828 | 0.0047 | Late embryogenesis abundant protein (LEA) family protein |
| 27 | AT5G40990 | 661.5888 | 140.8151 | 2.2321 | 0.0054 | GDSL LIPASE 1 (GLIP1). Component of plant resistance. Contains lipase signature motif and GDSL domain. Directly interferes with the fungal infection process by acting on fungal cell walls through its action as an antimicrobial compound. |
| 28 | AT4G26580 | 198.1982 | 43.9787 | 2.1721 | 0.0030 | RING/U-box superfamily protein |
| 29 | AT3G16920 | 2289.4576 | 518.709 | 2.142 | 0.0052 | CHITINASE-LIKE PROTEIN 2 is a chitinase-like protein expressed predominantly in stems. Mutants accumulate ligning in etiolated hypocotyls. |
| 30 | AT2G29130 | 492.9071 | 121.226 | 2.0236 | 0.0013 | LACCASE 2 is a putative laccase, knockout mutant had reduced root elongation under PEG-induced dehydration. |
| 31 | AT1G56660 | 776.2342 | 200.4403 | 1.9533 | 0.0047 | MAEBL domain protein |
| 32 | AT2G44810 | 2928.8644 | 760.5315 | 1.9453 | 0.0094 | DAD1 protein is a chloroplastic phospholipase A1 that catalyzes the initial step of jasmonic acid biosynthesis. |
| 33 | AT1G77525 | 40.2651 | 0 | Inf | 4.27E-05 | Defensin-like protein |
| 34 | AT2G30810 | 23.4409 | 0 | Inf | 0.0094 | Gibberellin-regulated family protein |
| 35 | AT2G38900 | 26.4996 | 0 | Inf | 0.0046 | Predicted to encode a PR (pathogenesis-related) peptide that belongs to the PR-6 proteinase inhibitor family. Six putative PR-6-type protein encoding genes are found in Arabidopsis: At2g38900, At2g38870, At5g43570, At5g43580, At3g50020 and At3g46860. |
| 36 | AT3G17600 | 18.8693 | 0 | Inf | 0.0096 | IAA31 shares several residues with the conserved domain II region, believed to act as a degron in many of the rapidly degraded Aux/IAA family members. An IAA31 fusion protein is quite long-lived, but can be degraded more rapidly in the presence of auxin. Unlike many other family members, IAA31 transcript levels do not rise in response to auxin. Nevertheless, overexpression of IAA31 leads to defects in auxin-related processes such as gravitropism, root development, shoot development, and cotyledon vascular development. |
| 37 | AT4G32490 | 23.1169 | 0 | Inf | 0.0081 | Early nodulin-like protein 4 (ENODL4). |
| 38 | AT5G38170 | 30.4322 | 0 | Inf | 0.0074 | Bifunctional inhibitor/lipid-transfer protein/seed storage 2S albumin superfamily protein |
| 39 | AT5G51105 | 59.9904 | 0 | Inf | 1.60E-07 | ECA1 gametogenesis family protein |
| 40 | AT5G38170 | 30.4322 | 0 | Inf | 0.0098 | Bifunctional inhibitor/lipid-transfer protein/seed storage 2S albumin superfamily protein |
| 41 | AT5G35935 | 0 | 88.9842 | inf | 0.0089 | Copia-like retrotransposon family |
| 42 | AT1G77870 | 0.8977 | 50.5554 | -5.8155 | 3.55E-05 | Membrane-anchored ubiquitin-fold protein 5 precursor (MUB5). |
| 43 | AT3G30720 | 52.6841 | 602.1716 | -3.5147 | 5.83E-08 | QQS is an orphan gene that arose recently in the Arabidopsis thaliana lineage. Overexpression of QQS in Arabidopsis increases protein content and decreases total starch content. Over expression of QQS in soybean, rice and maize also results in an increase in protein and decrease in starch levels suggesting that QQS affects similar pathways in a wide range of plants. QQS interacts with NF-YC4 in Arabidopsis and NF-YC4 homologs in rice, soybean and maize. In Arabidopsis QQS is localized the the cytoplasm and when complexed with NF-YC4 , it localizes to the nucleus. |
