# Supplementary material for: The protein elicitor Hrip1 enhances resistance to insects and early bolting and flowering in Arabidopsis thaliana
Source: PLoS One. 2019 Apr 25;14(4):e0216082. doi: 10.1371/journal.pone.0216082 (PMC6483360; doi:10.1371/journal.pone.0216082)
Supplement: S5 Table — (DOCX) [file pone.0216082.s009.docx]

| Gene name | Sequence | Description |
| --- | --- | --- |
| *Hrip1* | CATGCCATGGGCATGTACTTCTCGAATATACTCCCGG | pCAMBIA-1300 |
|  | GGGTCACCTCAGCACTGAGGCAAGTTACAGACC |  |
| AT1G65480 (*FT)* | GGTGGAGAAGACCTCAGGAA | qRT-PCR |
|  | GGTTGCTAGGACTTGGAACATC |  |
| AT2G44810 (*DAD1*) | TACGACGTCGAATCGTCAAT | qRT-PCR |
|  | GGTCGGGAAGTGGAAACTG |  |
| AT3G45140 (*LOX2*) | CTTACCCGCGGATCTCATC | qRT-PCR |
|  | ACTCCATGTTCTGCGGTCTT |  |
| AT5G42650 (*AOS*) | CACCGGCGTTAGTCAAATCT | qRT-PCR |
|  | CCGGCGGATTCTAAGAAAA |  |
| AT5G40990 *(GDSL1)* | ACATGACAGACGTTTTCAAGGA | qRT-PCR |
|  | TACGGTCCCGTGTTCAAGAT |  |
| AT2G38900 (*PR-6*) | TCGTTGTCAGAAACCCAACC | qRT-PCR |
|  | TGACCAACCAACCAACTATGC |  |
| AT3G18780 (*ACTIN2*) | GACCTTTAACTCTCCCGCTATG | qRT-PCR |
|  | TCACCAGAATCCAGCACAAT |  |
| AT5G09810 (*ACTIN7*) | GGCCGTTCTTTCTCTCTATGC | qRT-PCR |
|  | CCCTCGTAGATTGGCACAGT |  |
